# Arthropod-borne infections in travelled dogs in Europe

**DOI:** 10.1051/parasite/2013010

**Published:** 2013-03-12

**Authors:** Dietmar Hamel, Cornelia Silaghi, Kurt Pfister

**Affiliations:** Comparative Tropical Medicine and Parasitology, Veterinary Faculty, Ludwig-Maximilians-Universität München Leopoldstraße 5 80802 Munich Germany

**Keywords:** Vector-borne infections, Travelling dogs, Prophylaxis, Germany

## Abstract

Pet animal movement is ever increasing within the European Union and in that context canine vectorborne infections gained a considerable importance. Information on these infections in travelled dogs is nevertheless limited. A first prospective study on vector-borne infections was conducted in 106 dogs travelling from Germany to countries in South and South-East Europe. The dogs were screened prior to and consecutively up to three times after travel by haematological (Giemsa-stained buffy coat smears, Knott’s-Test), molecular biological (PCR) as well as serological (IFAT, DiroChek^®^-ELISA) methods for arthropod-borne infections. Seven animals were seropositive for antibodies against *Babesia canis* sspp., *Leishmania* spp. and/or *Ehrlichia canis* prior to travel to Italy, Spain, France, Croatia, Greece, or Hungary. In the consecutive screening after return there was no increase in the number of seropositive dogs. None was positive in direct methods. The mean duration of the stay was 17 days and 51% of the dogs were prophylactically treated with ectoparasiticidal formulations. Preliminary data from this study on canine vector-borne infections indicate a low risk for infection during a limited single stay in endemic countries.

## Abstract (fr)

Les mouvements des animaux de compagnie augmentent constamment dans l’Union européenne et dans ce contexte les infections canines transmises par les vecteurs prennent une importance considérable. Toutefois, les informations sur les infections des chiens qui voyagent sont limitées. Une première étude prospective a été conduite chez 106 chiens voyageant depuis l’Allemagne vers des pays du Sud et du Sud-est de l’Europe. Les chiens ont été testés avant et une à trois fois après le voyage par des tests hématologiques (frottis colorés au Giemsa, teste de Knott), moléculaires (PCR) et sérologiques (IFAT, DiroChek^®^-ELISA) des infections transmises par les vecteurs. Sept animaux étaient séropositifs pour les anticorps contre *Babesia canis* sspp., *Leishmania* spp. et/ou *Ehrlichia canis* avant leur voyage vers l’Italie, l’Espagne, la France, la Croatie, la Grèce ou la Hongrie. Dans les tests après leur retour il n’y avait pas d’augmentation du nombre de chiens séropositifs. Aucun n’a été positif par des méthodes directes. La durée moyenne du séjour était de 17 jours et 51 % des chiens ont été traités de manière prophylactique par des produits anti-ectoparasites. Les données préliminaires de cette étude sur les infections transmises par les vecteurs indiquent un faible risque d’infection pour un séjour unique de durée limitée en pays endémique.

## Introduction

Canine vector-borne infections are a steadily increasing field of veterinary research interest and their diagnosis, treatment and prophylaxis are of considerable importance in small animal medicine [5]. As travel restrictions within the European Union are essentially non-existent, animals are freely transferred from non-endemic regions into countries endemic for vector-borne infections and vice versa. A previous evaluation by questionnaire reported that more than 50% of 5,240 German dogs have crossed the border at least once and travelled e.g., to Italy, France and Spain [7]. Dogs originating from Central Europe can be considered as immunologically naïve to most vector-borne pathogens present in southern Europe and any sojourn to these regions sets dogs at risk of infection. However, recent studies evaluating laboratory data reported low seroprevalence rates (<5.0%) against *Babesia canis* spp., *Leishmania* spp. or *Ehrlichia canis* in travelled dogs [8, 10]. This may be indicative for a low exposure to infected vectors during a limited stay, although no information on prophylactic measures, the time or duration of travel was given in these studies [10, 24]. So far, no travelled dogs from Germany were tested involving the determination of a pre- and post-travel status on vector-borne infections. In this preliminary study, results on the serological, haematological and molecular biological examination of 106 dogs from Germany with sojourns into countries in the Mediterranean Basin and South-Eastern Europe are presented.

## Material and methods

In the spring 2009 and 2010 veterinarians were informed by an advertisement placed in the monthly magazine of the German Veterinarian Society (“Deutsches Tierärzteblatt”) about this prospective study. Interested veterinarians were provided with information on sampling time (e.g., for Knott’s-Test) and intervals of sampling. Pet owners presenting their dogs for a routine pre- and post-travel check-up/consultation and willing to participate were asked to fill out questionnaires. The testing (P0) one to two weeks prior to departure aimed at attaining an overview on existing infections, the testing intervals of two to four weeks (P1), of six to eight weeks (P2) and of six months (P3) after travel to detect babesiosis, hepatozoonosis and ehrlichiosis by direct pathogen detection and/or seroconversion (at P1), the prolonged interval of up to 6 months aimed at the detection of patent filarial infections (at P2-P3) and delayed seroconversion in case of *Leishmania* spp.-infection (at P1-P3) [1, 2, 9, 26, 27] (Table 1). Inclusion criteria were at least one pre- and one follow-up canine EDTA-blood sample (P0 and P1) available from routine screening. In total, 287 EDTA-blood samples from 106 dogs belonging to 91 different dog owners could be evaluated. The study was in compliance with local regulations.

**Table 1.** Pre- and post-travel screening panel for arthropod-borne infections.

Method	Timepoint of testing			
	P0	P1	P2	P3
Giemsa-stained blood smear	×	×		
Knott’s-Test	×	×	×	×
*Babesia canis canis*-PCR	×	×		
*Babesia canis vogeli*-PCR	×	×		
*Babesia gibsoni*-PCR	×	×		
*Hepatozoon canis*-PCR	×	×		
*Leishmania* spp.-PCR	×	×		
Filaria-PCR	×	×	×	×
*Ehrlichia canis*-PCR	×	×		
DiroChek^®^-ELISA	×	×	×	×
*Babesia canis*-IFAT	×	×		
*Leishmania*-IFAT	×	×	×	×
*Ehrlichia canis*-IFAT	×	×		

### Laboratory methods

Giemsa-stained buffy coat smears were performed for the detection of pathogens in the blood and Knott’s-Test for the detection of blood-borne microfilariae. The DiroChek^®^ Canine/Feline Antigen Test Kit (Synbiotics Corp., Missouri, USA) was used for screening for *D. immitis-*antigen. DNA was extracted from 200 μl EDTA-blood with the Qiagen DNA MiniKit (Qiagen, Hilden, Germany) following manufacturer’s instructions. Quality and quantity were tested with a spectrophotometer (NanoDrop^®^ ND-1000, Erlangen, Germany). Conventional PCRs were applied for the detection of *Babesia* spp.-, *H. canis*- and filarial-DNA (in case of positive Knott’s-Test) according to previously published protocols [3, 11, 23]. Real-time PCRs were used for the detection of *Leishmania* spp*.-* and *E. canis-*DNA [15, Silaghi et al., unpublished]. Each PCR run involved a positive control and a negative control (PCR-clean water). Plasma for IFAT was collected by centrifugation of EDTA-blood samples. Antibodies against *B. canis* spp. and *E. canis* were tested applying commercial IFAT-tests (MegaCor, Hörbranz, Austria) and an in-house *Leishmania* spp.-IFAT was used for the detection of anti-*Leishmania* spp.-antibodies [13]. Titres of 1:64 were considered as seroreactive to *B. canis* spp. and *Leishmania* spp. and of 1:40 to *E. canis*.

### Animal data and questionnaire

Participating pet owners were asked to fill in the provided questionnaires prior to and after travel to obtain individual animal data and information on travel destination, duration of the stay, prophylactic measures applied against arthropod vectors, on tick infestation on their dogs observed during holidays and to record any additional ectoparasiticidal treatment bought at travel destination.

## Results

### Laboratory results

#### Pre-travel screening (P0)

One hundred and six canine EDTA-blood samples were submitted for pre-travel screening in a mean of 7 days (1–47 days) prior to departure. None of the dogs was microfilaremic in Knott’s-Test or positive for *D. immitis-*antigen in DiroChek^®^-ELISA. All Giemsa-stained buffy coat smears were negative for parasites of the blood. Six dogs had antibodies against *B. canis* spp. (3×), *Leishmania* spp. (2×) and *E. canis* (1×) in IFAT, another animal was seropositive for both *B. canis* spp.- and *Leishmania* spp.-antibodies (Table 2). One animal positive for *B. canis* spp.-antibodies was vaccinated against canine babesiosis according to information given by the owner. All seropositive animals had travelled previously with their owners.

**Table 2.** Results of the screening for vector-borne infections in 106 travelling dogs.

Method	Timepoint of testing			
	P0	P1	P2	P3
Giemsa-stained blood smear	0/106	0/106		
Knott’s-Test	0/106	0/101^1^	0/60^1^	0/16
*Babesia canis canis*-PCR	0/106	0/106		
*Babesia canis vogeli*-PCR	0/106	0/106		
*Babesia gibsoni*-PCR	0/106	0/106		
*Hepatozoon canis*-PCR	0/106	0/106		
*Leishmania* spp.-PCR	0/106	0/106		
Filaria-PCR	n. t.^2^	n. t.	n. t.	n. t.
*Ehrlichia canis*-PCR	0/106	0/106		
DiroChek^®^-ELISA	0/106	0/106	0/61	0/16
*Babesia canis*-IFAT	4/106	2/106		
*Leishmania*-IFAT	3/106	3/106	2/61	1/16
*Ehrlichia canis*-IFAT	1/106	1/106		

1 Five (P1) and one (P2) blood sample(s) coagulated or of insufficient volume (<1 mL).

2 n. t. = not tested as all Knott’s-Tests were negative.

#### Post-travel screening (P1-P3)

One hundred and six samples were available for the first post-travel screening between four and 373 days (mean 38 days) after return. None of the samples was positive for vector-borne infections in direct methods. Dogs with antibodies against *Leishmania* spp (3×) and one seroreactive against *E. canis* were again tested positive. In two dogs, *B. canis* spp. IFAT was negative. A total of 61 samples were submitted between 34 and 423 days (mean 171 days) after return for P2 screening. There were neither microfilariae nor antigen of *D. immitis* detectable. Two animals were again seropositive for antibodies against *Leishmania* spp. while no sample was submitted from the third seroreactive dog. Only 16 samples out of 106 were sent in for a third post-travel examination (P3) between 193 and 493 days (mean 322 days) after the return to Germany. All were negative in Knott’s-Test and DiroChek^®^-ELISA. The two samples positive in P2 were again seropositive for *Leishmania* spp.-antibodies (Table 2).

### Animal data

The 44 male dogs and 62 bitches (106 dogs) in this study were between 0.5 and 14.5 years old (mean 5.1 years, standard deviation (*SD*) 3.4). Forty-four dogs of breed (26 different breeds), 31 mixed breed dogs and 31 dogs without specified breed were enrolled. Sixteen dogs were taken abroad previously, 90 dogs had not travelled across the German border. The travel duration was given for 98 dogs and varied between 3 and 62 days (mean 17 days, *SD* 8.6); 14 dogs were taken abroad for up to one week, 26 between 8 and 14 days, 40 between 15 days and 3 weeks and 18 for more than 3 weeks. The main travel period in 2009 was between May and September (89 dogs) and between July and September in 2010 (17 dogs). The travel destinations are summarized in Table 3. Pre-travel ectoparasiticidal treatment was performed according to information given by the pet owners in 54 dogs (51.0%) (28× deltamethrin [Scalibor^®^, Intervet]; 19× imidacloprid + permethrin [Advantix^®^, Bayer]; 3× permethrin [Exspot^®^, Essex]; 2× fipronil [Frontline^®^, Merial], 1× imidacloprid [Advantage^®^, Bayer]; 1× imidacloprid + moxidectin [Advocate^®^, Bayer]). Eleven dogs (10.3%) were administered other medication and no information was given in 41 (38.7%) cases. Tick infestation was observed by the owners on 7 treated (3× Advantix^®^, Bayer; 3× Scalibor^®^, Intervet; 1× Frontline^®^, Merial) and 3 untreated animals. Only 10 pet owners (11.0%) bought additional ectoparasiticidal products (5×; Scalibor^®^, Intervet; 4×; Advantix^®^, Bayer; 1× Frontline^®^, Merial) during holidays and seven (7.7%) pet owners bought other products.

**Table 3.** Travel destination by country and region.

Country (total dogs)	Region/province	Number of dogs
Italy (28)	Lazio	1
	Liguria	3
	Lombardia	5
	Sardinia	4
	South Tirol	3
	Tuscany	4
	Venetia	5
	No information/other	3
France (23)	Aquitania	2
	Bretagne	2
	Corsica	1
	Languedoc-Roussillon	8
	Medoc	1
	Pays de Loire	1
	Provence-Alpes-Côte d′Azur	7
	No information/other	1
Croatia (17)	Dalmatia	2
	Istria	7
	Primorje-Gorski County	7
	No information/other	1
Spain (17)	Alicante	1
	Balearic Islands	8
	Canary Islands	1
	Catalonia	3
	Costa Blanca	2
	Costa Brava	1
	No information/other	1
Greece (7)	Attica	1
	Corfu	1
	Makedonia	2
	Peloponnese	1
	South Aegean	1
	No information/other	1
Hungary (4)	Bács-Kiskum	2
	Somegy	1
	Vas	1
Roundtrip (10)	Mediterranean region/Balkans	10
Total		106

## Discussion

Canine populations from the Mediterranean Basin show high prevalence rates of vector-borne infections [24, 29]. Consequently, this topic attracted an increasing interest in veterinary medicine [5]. It is therefore noteworthy that in this study none of the dogs was infected with arthropod-borne infections due to the recent stay in South and South-East Europe. In total, seven dogs were seroreactive *to Leishmania* spp., *E. canis* and/or *B. canis* spp. at P0 prior to travel and all of these animals had been taken along on holidays previously. None of the animals were positive in Giemsa-stained buffy coat smears, Knott’s-Test, DiroChek^®^-ELISA or PCR. The overall seroprevalence rate of 6.6% in the 106 dogs is comparable to data presented in retrospective studies in dogs with travel history [8, 10]. A publication from the Netherlands based on questionnaire and diagnostic data identified a chance of 0.23% for individual travelling dogs for attaining a *Leishmania-*infection [28]. In a study from the UK, dogs with travel history and positive for leishmaniosis had spent at least six months in Spain, while the average stay in the present study was only 17 days [25]. The risk of acquiring a vector-borne infection is dependent on the presence of infected vectors, their seasonal activity, the duration of exposure, the presence of suitable (reservoir-)hosts and prophylactic treatment. As this study encompassed the summer holiday period in Germany, the time of travel did not necessarily coincide with peak activity of potential vectors. While the brown dog tick is active all year round in the subtropical areas of the Mediterranean, other vectors, e.g., phlebotomine sandflies, may show bimodal activity patterns in spring and autumn or primarily in the summer month, such as mosquitoes [4, 12, 26]. This impacts the chance of a potential pathogen transmission. The low number of examined blood samples may also bias the results of the study. Moreover, the diagnosis of vector-borne infections can vary considerably depending on the applied technique as well as of the course of clinical disease [17, 18]. Nevertheless, all animals were so far considered as healthy and it can be expected that dog owners would have sent additional EDTA-blood samples of their dogs in case of impaired health status. The results are in clear contrast to laboratory data from over 15 000 dogs imported into Germany published over the last ten years. Seroprevalence rates against *E. canis*, *Leishmania* spp. or *B. canis* spp. range from 10.6 to 18.5%, cumulative prevalence rates for microfilariae and *D. immitis*-antigen are 2.6% and 1.8% in over 10 000 dogs tested and 2.1% of 7500 dogs carry gamonts of *H. canis* in the blood [16, 22, 24]. Recommendations on prophylactic treatment are primarily based on data from studies in local dog populations from endemic countries [16, 29] Interestingly, of the 54 (51.0%) prophylactically treated dogs in this study, 50 (92.6%) were treated with actives (28× Scalibor^®^, Intervet; 19× Advantix^®^, Bayer; 3× Exspot^®^, Essex) registered for the use against *Phlebotomus* spp., giving evidence that veterinarians and pet owners are aware of this important vector and rely on formulations with anti-feeding effect. It has been proven that the prevalence of vector-borne infections increases with consecutive transmission seasons and with the age of dogs [6, 14]. A study in Italy conducted over two transmission seasons with more than 600 dogs presented an increase in *Leishmania*-antibody seroprevalence from 5% to more than 25% in untreated dogs while the rate in the deltamethrin-treated group remained at around 3% [14]. Moreover, the prophylactic treatment with imidacloprid/permethrin also significantly reduced the risk of infection in treated dogs exposed to high infection pressure [19–21]. Further studies should evaluate the impact of repeated travel in dogs to assess a cumulative risk for infection.

## Conclusion

These first preliminary results on vector-borne infections in travelled dogs present evidence for a low individual risk for infection during a single limited stay abroad. None of the prophylactically treated as well as untreated dogs attained an infection during the average stay of 17 days. Future studies should evaluate the impact on repeated sojourns in a larger cohort of animals.
